# Inflammatory gene expression in adipose tissue according to diagnosis of anxiety and mood disorders in obese and non-obese subjects

**DOI:** 10.1038/s41598-018-35759-9

**Published:** 2018-11-30

**Authors:** Leticia Coín-Aragüez, Francisco Javier Pavón, Alba Contreras, Adriana-Mariel Gentile, Said Lhamyani, Yolanda De Diego-Otero, Yolanda Casado, Wilfredo Oliva Olivera, Gabriel Olveira, Francisco J. Tinahones, Lucía Pérez Costillas, Rajaa El Bekay

**Affiliations:** 1 Unidad de Gestión Clínica Endocrinología y Nutrición, Instituto de Investigación Biomédica de Málaga (IBIMA), Complejo Hospitalario de Málaga (Virgen de la Victoria), Universidad de Málaga, CIBER de Fisiopatología de la Obesidad y Nutrición (CIBERobn), Instituto de Salud Carlos III, Málaga, Spain; 2 Unidad de Gestión Clínica de Salud Mental, Instituto de Investigación Biomédica de Málaga (IBIMA), Hospital Regional Universitario de Málaga, Universidad de Málaga, Málaga, Spain; 3 IBIMA, Universidad de Málaga, Facultad de Ciencias, Campus Teatinos s/n – 29071, Málaga, Spain; 4 UGC Endocrinología y Nutrición, Instituto de Investigación Biomédica de Málaga (IBIMA), Hospital Universitario Regional de Málaga, Universidad de Málaga, CIBERDEM CB07/08/0019, Instituto de Salud Carlos III, Málaga, Spain; 5 Unidad de Gestión Clínica Endocrinología y Nutrición, Instituto de Investigación Biomédica de Málaga (IBIMA), Hospital Regional Universitario de Málaga, Universidad de Málaga, CIBER de Fisiopatología de la Obesidad y Nutrición (CIBERobn), Instituto de Salud Carlos III (ISCIII), Málaga, Spain

## Abstract

Psychiatric disorders have been widely reported to be associated with systemic inflammation upregulation and adiposity. However, there are no data that link adipose tissue inflammation to these mental disorders. The analysis of adipokines and inflammation-related markers in adipose tissue could help to elucidate the potential association between obesity and mental health. An observational study was conducted in samples of patients consisting of non-obese and obese subjects, who were diagnosed with anxiety or mood disorders. Gene expression of adiponectin (*ADIPOQ*), leptin (*LEP*) and inflammatory markers (*IL6, IL1B, TNF, CCL2, CSF3, ITGAM*, and *PLAUR*) were determined in visceral (VAT) and subcutaneous (SAT) adipose tissues. Our results showed that the gene expression of adipokines and inflammation-related markers was higher in the VAT and SAT of obese subjects compared with non-obese subjects. Regarding mental disorders, all the inflammatory genes in the VAT were significantly higher in non-obese subjects with anxiety or mood disorders than in subjects without mental disorders, except for *TNF* and *ITGAM*. Additionally, *IL6* expression was significantly lower in SAT. In contrast, obese patients diagnosed with anxiety or mood disorders only showed significantly lower expression levels of *IL1B* in VAT and *ADIPOQ* in SAT when compared with obese subjects without mental disorders. These data suggest the potential involvement of VAT inflammation in anxiety and mood disorders, involving complex mechanisms which are strongly affected by obesity.

## Introduction

Currently, obesity represents a major priority for the public health agenda and its prevalence is alarming^1^. Obesity is a major risk factor for metabolic syndrome and type 2 diabetes, which comprise disease conditions such as cardiovascular, musculoskeletal, and psychiatric disorders^2–5^. In this sense, the presence of psychiatric disorders is common in those suffering from obesity. Therefore, the high rate of comorbidity includes not only medical, but also mental conditions, and efforts need to be taken to control its increase. Mental disorders, in concordance with the current state of obesity and metabolic syndrome, are expected to reach epidemic proportions worldwide between 2016 and 2050. Among them, depressive disorders are a significant contributor to the global burden of these disorders and affects 350 million people worldwide^6^. There have been several prospective epidemiological reports evaluating the relationship between body mass index (BMI) and psychiatric disorders. In fact, it has been suggested that obesity and associated factors could play a role in the development of psychiatric disorders^7^. Thus, it has been found that obesity increases the risk of developing depression, and conversely, depressive disorders were found to be predictive of developing obesity^4,7,8^. Other studies have shown that obesity is positively associated with anxiety^9^, and that psychosocial stress influences the development of adiposity^10,11^.

Evidence has suggested that inflammation may provide a mechanistic link between adiposity and mental disorders, and a low-grade inflammatory state is observed in obese subjects but also in depression, psychosis and other major psychiatric disorders. In adipose tissue, local inflammation is produced as a consequence of adipocyte hypertrophy, leading to an increased secretion of cytokines and chemokines together with an altered pattern of secreted adipokines^11–15^. An incompetent and dysfunctional adipose tissue is being described to be as a critical risk factor for the onset of obesity-associated metabolic complications^12,13^. Accordingly, increased proinflammatory cytokine levels have been also found in patients with neurodegeneration and mood disorder symptoms such as anhedonia, depressed mood, and lethargy^4,14^.

Although adipose tissue serves as an energy depot, visceral (VAT) and subcutaneous adipose tissues (SAT) are different in the sense that physiological and morphological aspects might be crucial in the metabolic homeostasis^15^. In fact, excessive VAT and SAT are key contributors to abdominal obesity but differ in their location, structural composition, metabolic activity and functional significance^16^. While VAT is strongly associated with insulin resistance, atherosclerosis and dyslipidemia in obese subjects, SAT demonstrates no association with these adverse obesity phenotypes. Furthermore, it has been also proposed that in deleterious conditions, VAT produces more pro-inflammatory molecules than SAT^15,17^. In addition to inflammatory mediators such as cytokines, adiponectin and leptin are two adipokines that are mainly secreted by adipose tissue and are known to play a pivotal role in the pathophysiology of obesity and associated metabolic disorders^18^. They have also been described to participate in the pathophysiology of psychiatric disorders such as major depression and anxiety-like disorders^19,20^.

In spite of all these investigative efforts, the inflammatory mechanisms underlying the link between obesity and mental disorders have not yet been characterized in the adipose tissue. Most studies have been limited to the analysis of proinflammatory mediators (i.e., cytokines) and adipokines in the plasma/serum of obese subjects or patients with mental disorders. In order to establish an association between the expression of inflammatory markers within adipose tissue and the presence of mental disorders in obesity, we examined the gene expression of relevant inflammatory markers and adipokines within adipose tissue in obese and non-obese subjects according to the diagnosis of anxiety and mood disorders. Through the present observational study, we have performed a description and analysis of variables and confounders, and differences in the gene expression of inflammatory markers and adipokines in the VAT and SAT were found in obese and non-obese subjects with mental disorders. However, further research is needed to establish the causality between alterations in inflammatory-associated markers in the adipose tissue and the presence of psychiatric disorders.

## Results

### Clinical and biochemical characteristics in patients according to obesity

We explored the clinical and biochemical characteristics based on the obesity status. Whereas the non-obese group had a mean BMI of 25.0 ± 2.5 kg/m^2^, the obese group had a mean BMI of 46.6 ± 12.1 kg/m^2^. As shown in Table 1, no differences in mean age (56–57 years-old) or sex (36–45% women) were observed between both groups. Regarding biochemical parameters, we observed significant differences in several variables related to energy metabolism and inflammation in the blood. Thus, HOMA-IR and glucose levels were higher in the obese group (*P* < 0.001 and *P* < 0.05, respectively) compared with the non-obese group, but HDL levels were lower (*P* < 0.001).

**Table 1:** Clinical and biochemical characteristics according to the presence of obesity.

Variables	Non-obese group (BMI < 30)	Obese group (BMI ≥ 30)	*P*-value
	*n* = 56	*n* = 53	
BMI (kg/m^2^) [mean ± SD]	25.18 ± 2.50	46.65 ± 12.14	**<0.001** ^**a**^
Sex (females) [n (%)]	25 (44.6)	19 (35.8)	0.350^b^
Age (years) [mean ± SD]	57.38 ± 14.04	56.38 ± 12.95	0.701^a^
HOMA-IR [mean ± SD]	2.852 ± 2.117	4.823 ± 2.924	**<0.001** ^**a**^
Glucose (mg/dL)[mean ± SD]	97.65 ± 24.54	103.66 ± 18.52	**0.023** ^**a**^
Total cholesterol (mg/dL) [mean ± SD]	209.16 ± 38.10	207.35 ± 43.25	0.822^a^
HDL (mg/dL) [mean ± SD]	56.62 ± 15.15	46.91 ± 11.84	**<0.001** ^**a**^
LDL (mg/dL) [mean ± SD]	126.18 ± 30.03	131.46 ± 30.45	0.387^a^
Triglycerides (mg/dL) [mean ± SD]	124.53 ± 75.13	148.78 ± 85.41	0.123^a^
IL1β (pg/mL) [mean ± SD]	0.065 ± 0.104	0.096 ± 0.138	0.117^a^
IL6 (pg/mL) [mean ± SD]	1.820 ± 1.613	4.306 ± 4.371	**0.002** ^**a**^
TNFα (pg/mL) [mean ± SD]	2.121 ± 0.808	3.246 ± 1.423	**0.011** ^**a**^

^a^*P*-value from Student t-test or Mann-Whitney U-test.

^b^*P*-value from Fisher’s exact test or Chi-square test.

Abbreviations: SD, standard deviation; BMI, body mass index; HOMA-IR, homeostatic model assessment of insulin resistance; HDL, high-density lipoprotein; LDL, low-density lipoprotein; IL, interleukin; TNF, tumor necrosis factor.

Serum IL-6 and TNFα levels were significantly higher in the obese group (*P* < 0.05) than in the non-obese group. Consistently, correlation analyses showed a significant and positive association of BMI with IL-1β (r = 0.356, *P* = 0.036), IL-6 (r = 0.473, *P* = 0.004) and TNFα (r = 0.375, *P* = 0.032) levels.

### Gene expression of adipokines and inflammatory markers in the adipose tissue of patients according to obesity

As a first approximation, a multiple correlation analysis between mRNA levels of these genes and BMI values was performed in the total sample. Overall, we observed positive and significant correlations between both adipose tissues, particularly in the VAT of obese participants (Table S1).

After confirming the association with BMI, the effects of obesity on mRNA levels of these genes were evaluated in the VAT and SAT using analysis of covariance(ANCOVA) with ‘obesity status’ (non-obese and obese groups) and ‘sex’ as factors and controlling for ‘age’. Data were examined for normality and all of the mRNA levels were log-transformed for analysis. Back-transformed marginal means on the original scale are shown in Table 2.

**Table 2:** Gene expression of adipokines and inflammatory mediators in the adipose tissue according to obesity.

*Gene* (Protein) [mean and 95% CI]	VAT			SAT		
	Non-obese group (BMI < 30)	Obesegroup (BMI ≥ 30)	*P*-value^a^	Non-obese group (BMI < 30)	Obesegroup (BMI ≥ 30)	*P*-value^a^
	*n* = 56	*n* = 53		*n* = 56	*n* = 53	
*ADIPOQ* (Adiponectin)	27.86 (20.51–37.84)	15.49 (11.22–21.33)	**0.010**	24.43 (18.03–33.04)	30.83 (22.44–42.36)	0.294
*LEP* (Leptin)	0.333 (0.220–0.502)	0.698 (0.453–1.074)	**0.015**	0.89 (0.63–1.24)	1.76 (1.25–2.48)	**0.005**
*IL1B* (Interleukin 1 Beta/IL1β)	0.044 (0.026–0.074)	0.184 (0.107–0.315)	**<0.001**	0.076 (0.051–0.113)	0.147 (0.097–0.223)	**0.024**
*IL6* (Interleukin 6/IL6)	0.010 (0.005–0.018)	0.032 (0.017–0.061)	**0.009**	0.017 (0.009–0.033)	0.022 (0.011–0.044)	0.597
*TNF* (Tumor Necrosis Factor Alpha/TNFα)	0.019 (0.011–0.033)	0.044 (0.025–0.079)	**0.041**	0.024 (0.014–0.041)	0.068 (0.039–1.19)	**0.009**
*CCL2* (Chemokine (C-C motif) Ligand 2/CCL2)	0.297 (0.201–0.441)	0.757 (0.502–1.140)	**0.001**	0.412 (0.305–0.557)	0.714 (0.521–0.979)	**0.014**
*CSF3* (Colony Stimulating Factor 3/CSF3)	2.37 (1.26–4.47) × 10^−4^	15.42 (7.94–29.92) × 10^−4^	**<0.001**	4.95 (2.36–10.42) × 10^−4^	24.32 (11.17–52.97) × 10^−4^	**0.004**
*ITGAM* (Integrin, Alpha M/ITGAM)	0.538 (0.396–0.731)	0.662 (0.480–0.910)	0.359	0.232 (0.169–0.318)	0.558 (0.401–0.778)	**<0.001**
*PLAUR* (PlasminogenActivator, Urokinase Receptor/PLAUR)	0.070 (0.051–0.096)	0.129 (0.100–0.191)	**0.004**	0.066 (0.050–0.086)	0.125 (0.094–0.165)	**0.001**

Data are indicated as mean and 95% confidence interval.

^a^*P*-value from ANCOVA for *Obesity status* factor.

Abbreviations: CI, confidence interval; VAT, visceral adipose tissue; SAT, subcutaneous adipose tissue; BMI, body mass index.

#### Visceral adipose tissue

We found a significant main association of ‘obesity status’ with mRNA levels of adiponectin (*F*_1,102_ = 6.890*, P* = 0.010), leptin (*F*_1,102_ = 6.062*, P* = 0.015), IL-1β (*F*_1,102_ = 14.327*, P* < 0.001), IL-6 (*F*_1,102_ = 7.191*, P* = 0.009), TNFα (*F*_1,102_ = 4.271*, P* = 0.041), PLAUR (*F*_1,102_ = 8.848, *P* = 0.004), CCL2 (*F*_1,102_ = 10.667*, P* = 0.001) and CSF3 (*F*_1,102_ = 16.403*, P* < 0.001). In fact, there were higher mRNA levels in the obese group relative to the non-obese group, except for adiponectin. However, we observed no association of ‘sex’ or interaction of ‘obesity status’ and ‘sex’ with mRNA levels of these adipokines and inflammatory markers in the VAT.

#### Subcutaneous adipose tissue

Multivariate ANCOVA revealed a significant main association of ‘obesity status’ with mRNA levels of leptin (*F*_1,102_ = 8.089*, P* = 0.005), IL-1β (*F*_1,102_ = 5.217*, P* = 0.024), TNFα (*F*_1,102_ = 7.150*, P* = 0.009), CCL2 (*F*_1,102_ = 6.227*, P* = 0.014), CSF3 (*F*_1,102_ = 8.593*, P* = 0.004), ITGAM (*F*_1,102_ = 14.416*, P* < 0.001) and PLAUR (*F*_1,102_ = 10.832*, P* = 0.001). Specifically, mRNA levels of leptin and inflammatory markers in the SAT were higher in the obese group than in the non-obese group. Similarly to VAT, we found no association of ‘sex’ or interaction between factors with mRNA levels of these mediators.

#### Correlation between visceral and subcutaneous adipose tissues

We conducted a secondary analysis to investigate the correlation between mRNA levels in VAT and SAT (Table S2). There was a positive and significant association between leptin mRNA levels and all of the inflammatory markers in the total sample. However, differences in these associations between VAT and SAT were detected in the non-obese and obese groups. While non-obese patients showed a significant correlation for leptin (*P* < 0.001), IL-1β (*P* < 0.001), IL-6 (*P* < 0.001) and TNFα (*P* < 0.05), obese patients showed a significant correlation for IL-6 (*P* < 0.01), ITGAM (*P* = 0.001) and PLAUR (*P* < 0.01).

### Anxiety and mood disorders in patients according to obesity

Nearly 60% of patients were diagnosed with anxiety (*n* = 43) [age 59.1 (SD = 13.3) years-old] or mood (*n* = 22) [age 56.1 (SD = 13.6) years-old] disorders using the structured ‘International Mini Neuropsychiatric Interview’ (MINI). There were differences in the mean BMI of obese patients according to the presence of these mental disorders. Obese patients with anxiety or mood disorders had lower BMI than those patients without mental disorders [41.81 (95% CI = 37.61–46.01) kg/m^2^ and 55.30 (95% CI = 52.38–58.22) kg/m^2^, respectively]. In contrast, the non-obese group had similar mean BMI [25.11 (95% CI = 24.13–26.09) kg/m^2^ and 25.27 (95% CI = 24.31–26.23) kg/m^2^, respectively].

### Gene expression of adipokines and inflammatory markers in the adipose tissue of non-obese and obese patients according to anxiety and mood disorders

Because mRNA levels of adipokines and inflammatory mediators were strongly associated with ‘obesity status’, we evaluated their association with anxiety and mood disorders separately for non-obese and obese patients. Two-way ANCOVA was used with ‘mental disorder status’ [non-mental disorders (non-MD) and anxiety/mood disorders (A/MD) groups] and ‘sex’ as factors and controlling for ‘BMI’, ‘age’ and ‘psychiatric medication’. Subsequently, mRNA levels of those genes which were significantly associated with ‘mental disorder status’ were again evaluated to distinguish between anxiety and mood disorders using ‘type of mental disorder’ as a factor [non-MD, anxiety disorders (ANX) and mood disorders (MOOD) subgroups].

**Figure 1:**
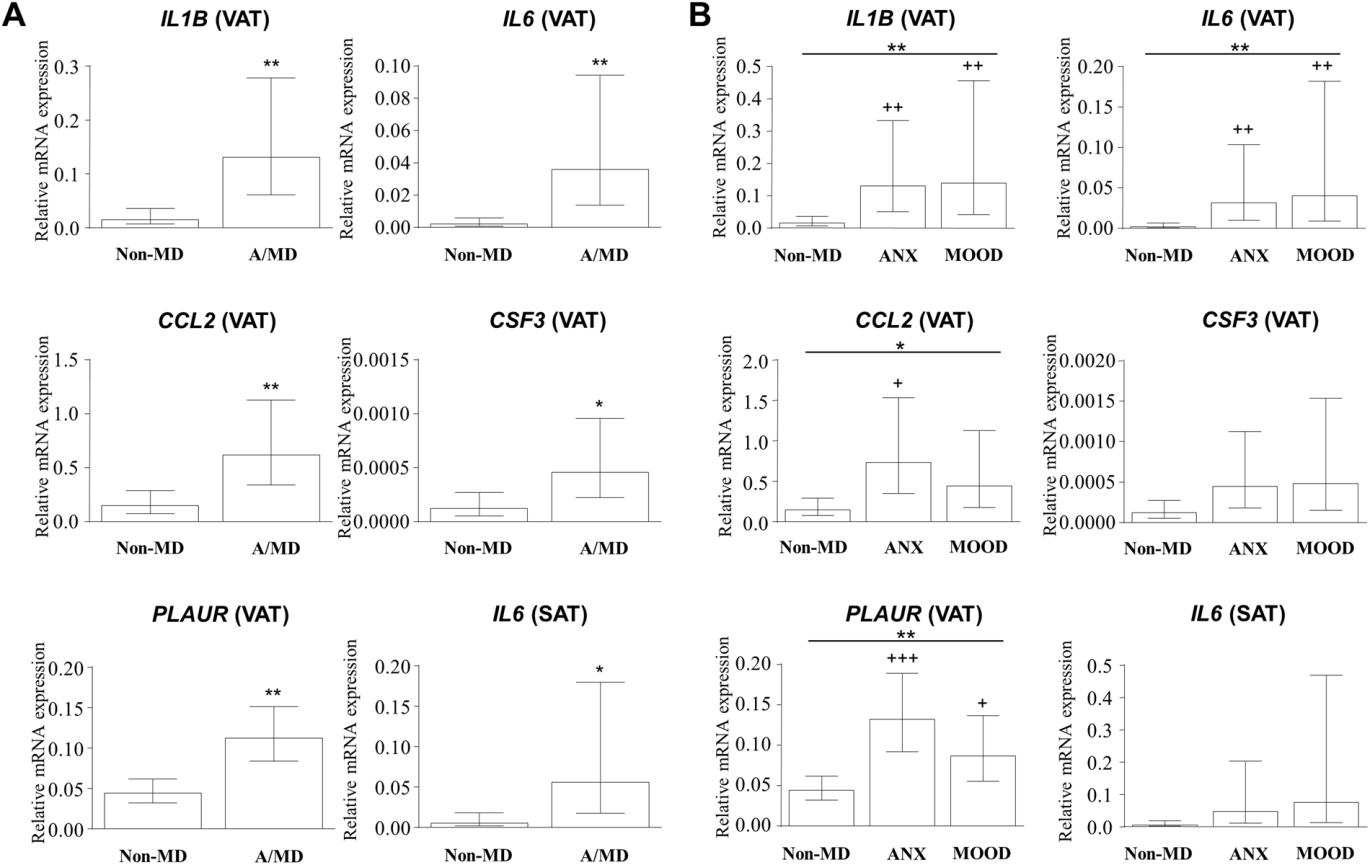
Gene expression of inflammatory markers altered in the adipose tissue of the non-obese group according to anxiety and mood disorders. Relative mRNA levels of *IL1B, IL6, CCL2, CSF3* and *PLAUR* in visceral adipose tissue and *IL6* in the subcutaneous adipose tissue according to ‘mental disorder status’ (**A**) or ‘type of mental disorder’ (**B**). Bars are estimated marginal means and 95% CI. *P < 0.05 and **P < 0.01 denotes significant main effect using ANCOVA. (+) *P* < 0.05, (++) *P* < 0.01 and (+++) *P* < 0.001 denotes significant differences compared with the non-MD subgroup. Abbreviations: VAT, visceral adipose tissue; SAT, subcutaneous adipose tissue; Non-MD, non–mental disorder subgroup; A/MD, anxiety/mood disorders subgroup; ANX, anxiety disorders subgroup; MOOD, mood disorders subgroup.

**Figure 2:**
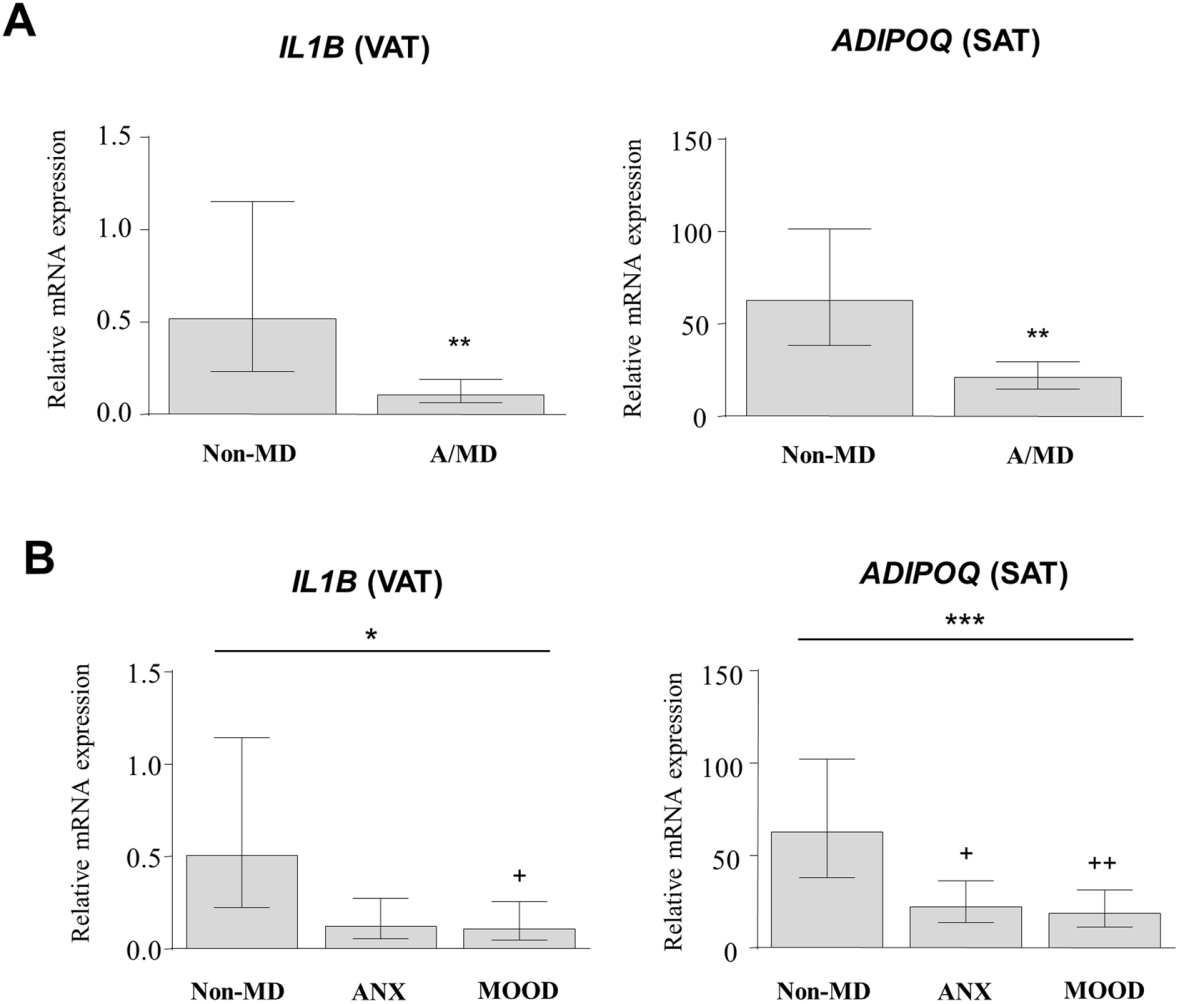
Gene expression of IL-1β and adiponectin in the adipose tissue of the obese group according to anxiety and mood disorders. Relative mRNA levels of *IL1B* in visceral adipose tissue and *ADIPOQ* in subcutaneous adipose tissue according to ‘mental disorder status’ (**A**) or ‘type of mental disorder’ (**B**). Bars are estimated marginal means and 95% CI. *P < 0.05 and **P < 0.01 and ***P < 0.001 denotes significant main effect using ANCOVA. (+) *P* < 0.05 and (++) *P* < 0.01 denotes significant differences compared with the non-AMD subgroup. Abbreviations: VAT, visceral adipose tissue; SAT, subcutaneous adipose tissue; Non-MD, non–mental disorders subgroup; A/MD, anxiety/mood disorders subgroup; ANX, anxiety disorders subgroup; MOOD, mood disorders subgroup.

All of the mRNA levels were log-transformed for analysis and estimated marginal means were presented in Figs 1 and 2 after back-transformation.

#### Non-obese group

The composition of the non-obese group by sex was 9 female and 16 male patients with non-mental disorders, and 22 female and 9 male patients diagnosed with anxiety/mood disorders. Overall, we observed a significant main association of anxiety and mood disorders with mRNA levels of inflammatory mediators in the adipose tissue of non-obese patients, particularly in the VAT (Fig. 1). However, we found no differences in mRNA levels of adipokines.

As shown in Fig. 1A, ANCOVA revealed a significant association of ‘mental disorder status’ with mRNA levels of IL-1β (*F*_1,47_ = 10.885*, P* = 0.002), IL-6 (*F*_1,47_ = 12.004*, P* = 0.001), CCL2 (*F*_1,47_ = 7.802*, P* = 0.008), CSF3 (*F*_1,47_ = 4.518*, P* = 0.039) and PLAUR (*F*_1,47_ = 13.423*, P* = 0.001) in the VAT, and mRNA levels of IL-6 (*F*_1,47_ = 6.046*, P* = 0.018) in the SAT. In all these cases, non-obese patients with anxiety/mood disorders (A/MD subgroup) showed significantly higher mRNA levels of these genes relative to non-obese patients without mental disorders (non-MD subgroup). In contrast, there were no associations of ‘sex’ or interaction between factors with mRNA levels of these mediators.

With respect to the distinction between anxiety and mood disorders (Fig. 1B), we found a main association of ‘type of mental disorder’ with mRNA levels of IL-1β (*F*_2,45_ = 5.212*, P* = 0.009), IL-6 (*F*_2,45_ = 5.458*, P* = 0.008), CCL2 (*F*_2,45_ = 4.076*, P* = 0.024) and PLAUR (*F*_1,47_ = 7.996*, P* = 0.001) in the VAT. *Post hoc* comparisons revealed that non-obese patients diagnosed with anxiety or mood disorders (A/MD subgroup) had significantly higher mRNA levels of IL-1β (*P* < 0.01), IL-6 (*P* < 0.01) and PLAUR (*P* < 0.001 and *P* < 0.05, respectively) than non-obese patients without mental disorders (non-MD). For CCL2, we observed that only patients with anxiety disorders (ANX subgroup) had significantly (*P* < 0.05) higher mRNA levels.

#### Obese group

The composition of the obese group by sex was 11 female and 8 male patients with non-mental disorders, and 23 female and 11 male patients diagnosed with anxiety/mood disorders. In the obese group, there were significant associations of anxiety and mood disorders with mRNA levels of inflammatory mediators displaying a lower expression, except for IL-1β in the VAT (Fig. 2).

As shown in Fig. 2A, ANCOVA revealed a main association of ‘mental disorder status’ with mRNA levels of IL-1β (*F*_1,46_ = 8.726*, P = *0.005) and adiponectin (*F*_1,46_ = 11.663*, P* = 0.001) in VAT and SAT, respectively. Thus, IL-1β and adiponectin mRNA levels were significantly lower in obese patients with anxiety/mood disorders (A/MD subgroup) than in obese patients without mental disorders (non-MD subgroup). Similarly to the non-obese group, there were no significant associations of ‘sex’ or interaction between factors with mRNA levels of these genes.

When we separated anxiety and mood disorders (Fig. 2B), ANCOVA showed a main association of ‘type of mental disorder’ with mRNA levels of IL-1β (*F*_2,44_ = 3.722*, P* = 0.032) and adiponectin (*F*_2,44_ = 5.834*, P* = 0.006). Pairwise comparisons indicated that obese patients diagnosed with anxiety disorders (ANX subgroup) had significantly lower adiponectin mRNA levels (*P* < 0.05) than in non-obese patients without mental disorders (non-MD subgroup), whereas obese patients with mood disorders (MOOD subgroup) had lower mRNA levels of IL-1β (*P* < 0.05) and adiponectin (*P* < 0.01).

Because we found lower IL-1β mRNA levels in obese patients with anxiety/mood disorders (A/MD subgroup) than in patients without mental disorders (non-MD subgroup), we measured the protein expression of IL-1β in the blood. Unlike what we found in the VAT, we observed higher serum IL-1β levels in obese patients with anxiety or mood disorders than in the non-MD subgroup (0.046 ± 0.080 and 0.132 ± 0.055 pg/mL, respectively) after controlling for BMI. In addition to IL-1β, IL-6 and TNFα serum levels were higher in this obese subgroup (IL-6: 3.54 ± 4.10 vs. 2.90 ± 1.41 pg/mL; and TNFα: 2.96 ± 1.27 vs. 2.38 ± 1.48 pg/mL; respectively).

## Discussion

There is increasing evidence that obesity, anxiety and depression are comorbid disorders^21,22^. Because adipose tissue dysfunction could be involved in the development of psychiatric disorders, previous studies have examined the relationship between adiposity and mental health using markers of inflammation in the blood^4,19,23^. Accordingly, we showed that obese subjects have higher serum cytokine levels compared with non-obese subjects, particularly in obese patients with anxiety or mood disorders. However, the aim of the present study was to describe and analyze the potential association between the gene expression of inflammatory markers (and adipokines) in adipose tissue and the presence of prevalent psychiatric disorders (i.e., anxiety and mood disorders) in obese and non-obese patients.

The main findings of this observational study were as follows: (1) The gene expression of inflammation-related markers and adipokines was higher in the VAT and SAT of obese patients with respect to non-obese patients, which was in line with the strong and positive correlation between mRNA levels of these mediators and BMI in both adipose tissues; (2) The gene expression of relevant inflammation-related markers (*IL1B, IL6, CCL2, CSF3* and *PLAUR*) were significantly higher in the VAT of non-obese subjects with anxiety or mood disorders, whereas only *IL6* expression was higher in the SAT among non-obese patients without mental disorders; (3) In contrast, obese patients diagnosed with anxiety or mood disorders only showed significantly lower *IL1B* levels in the VAT and *ADIPOQ* in the SAT relative to obese subjects without mental disorders.

Therefore, while the obesity status was associated with higher expression of inflammatory markers in both adipose tissues, mood and anxiety disorders were associated with higher gene expression of inflammatory markers in the VAT of non-obese patients but not in obese patients. These results in non-obese patients, suggest that VAT could be more sensitive than SAT to changes in the inflammatory response linked to mental disorders. According to our results, studies in women have shown that VAT is related to mood disorders, and depressive symptoms are indeed associated with increased inflammation in this type of adipose tissue^24^. Moreover, emerging evidence indicates that VAT may impair resilience to stress, and therefore differentially impact the subsequent risk of mood and/or anxiety disorders^25^.

Mental disorders such as depressive disorders and anxiety have been suggested to be related to systemic inflammation upregulation^4,26^. In fact, patients with major depression have increased levels of inflammatory markers and cytokines in the blood^14^. Increasing evidence indicates that one possible important source of inflammation in mood disorders could be adiposity^27^ assuming that adipose tissue is a known source of inflammatory factors including adipokines, chemokines, and cytokines^28^. On the other hand, it is well known that macrophages infiltrate into white adipose tissue^29,30^ and this contributes to the production of inflammatory factors such as chemokines and the development of some morbidities such as obesity and metabolic alterations. Interestingly, our data suggest that inflammation modulated by IL-1β, IL-6, PLAUR, and CCL2 in the VAT could be related to anxiety and mood disorders as long as patients are not in an obesity state. Several studies are in agreement and have shown that these proinflammatory factors are higher in serum from subjects with psychiatric disorders and are involved in the progression and severity of depressive and anxiety disorders^26,31^. Cytokines such as IL-1β and IL-6 have been found to have neuroendocrine activity and induce behavioral changes related to anxiety^32^, including preclinical models^33^. Thus, IL-1β contributes to psychological stress responses and has been implicated in several psychiatric disorders, most notably in major depressive disorders, including a specific behavioral complex, sickness behavior, which is characterized by sleep disorders, anxiety, and diminished social interactions^34^. Also, a preclinical study has shown that IL-1β modulates anxiety and fear-related behaviors^35^. Regarding IL-6, this cytokine has been reported to be higher in depressed subjects than in non-depressed subjects^31,36^, which is in line with our results since IL-6 mRNA levels were altered in subjects with both anxiety and mood disorders. In addition to cytokines, both CCL2 and PLAUR mRNA levels were found to be higher in non-obese patients with mental disorders. Other studies have also shown that CCL2 levels were significantly higher in patients with major depression and in female patients with stress-related mental disorders^37,38^.

Regarding obese patients, the relationship between inflammatory markers and mental disorders was no observed, with the exception of IL-1β. From our point of view, this lack of differences in the gene expression of inflammatory markers in the adipose tissue could be the consequence of an exacerbated obesity-induced inflammation and, therefore this excessive inflammatory response could prevent from detecting the effects of these mental disorders. However, this overlapping effect of obesity is mere speculation because the causality between alterations of inflammatory mediators and obesity or mental disorders cannot be established in an observational study such as this one. The only significant differences observed in the obese group with respect to inflammatory factors were in IL-1β mRNA levels. Thus, the *IL1B* expression in the VAT was lower in obese patients with mood disorders than in those patients without mental disorders. This surprising finding was confirmed after including BMI as a cofactor for the statistical analysis of IL-1β mRNA levels in obese patients according to the presence of mental disorders. Moreover, lower IL-1β mRNA levels were also observed in obese patients with mood and/or anxiety disorders after selecting subjects with comparable BMI values (data not shown).

Our results show that the gene expression of adiponectin was the only adipokine that was affected by anxiety or mood disorders resulting in lower mRNA levels in the SAT of obese patients with anxiety and mood disorders. These findings are concordant with the fact that adiponectin activity has been well-described to be strongly associated with several mental disorders^39^. Thus, decreased serum adiponectin levels have been also reported in major depressive disorders, panic disorders, and schizophrenia^23,40,41^. Furthermore, it has been described that adiponectinemia reduction is related to major depression severity^42^. Other studies have suggested that adiponectin levels are critical for the determination of susceptibility to depressive behaviors and that adiponectin could be a potential therapeutic target for the treatment of depression^43^.

These data indicate that it is relevant to study how adipose tissue inflammation could be associated with the presence of anxiety and mood disorders in both obese and non-obese subjects since there is evidence of a bidirectional link between obesity or metabolic alterations and neuropsychiatric status. In this regard, various studies converge to show that inflammatory processes originating from the adipose tissue environment spread to the brain where they lead to substantial changes in neuroendocrine circuitry activity, neurotransmitter metabolism, and signaling, and also in neurogenesis. Together, these alterations contribute to shape the propitious bases for the development of obesity-related neuropsychiatric comorbidities^4^. In addition, there is neuroanatomical and functional evidence for the sympathetic nervous system innervation of white adipose tissue, which has at least three functions: lipolysis, regulation of fat cell number and control of some white adipose tissue-secreted proteins^44,45^. Consequently, the present results and previously published data, point to the necessity of great efforts to elucidate the mechanisms behind the cross-talk between brain and adipose tissue and subsequently between obesity and mental disorders.

### Limitations

Despite the efforts in the design phase and selection of patients, and the use of appropriate statistical analyses, we are aware of the limitations of this observational study. (1) A main disadvantage is that the causality between alterations in the gene expression of inflammatory mediators/adipokines in adipose tissue and obesity or mental disorders cannot be established. (2) The validity of the present results with a relatively small sample size has to be proven in a larger cohort of patients. (3) A longitudinal study is needed to evaluate the gene expression of inflammatory factors and adipokines in both adipose tissues according to the variation in the BMI of the same patients with or without these mental disorders. Complementarily, preclinical studies will be able to elucidate the mechanisms underlying the reciprocal relationship between obesity and psychiatric disorders. (4) There are numerous socio-demographic and physiological variables that could influence the expression of these genes in adipose tissue, and the inclusion of potential confounding variables and covariates in the present statistical analyses has important restrictions related to the sample size to avoid overfitting. For example, although the use of psychotropic medications was controlled, there is great diversity in the use of other drugs such as statins (particularly in obese patients) that should be kept in mind. (5) Finally, we cannot discard the effects of other common psychiatric disorders (e.g., psychotic and personality disorders) that were not considered in the MINI interview.

## Materials and Methods

### Patients and recruitment

The present observational study was conducted in 109 patients waiting for bariatric or abdominal laparoscopic surgery at the Hospital Regional Universitario de Málaga and Hospital Universitario Virgen de la Victoria (Málaga, Spain), who were psychiatrically assessed.

The procedure for recruiting was non-random convenience sampling according to the participation criteria and medical records. Therefore, the initial cohort consisted of 120 subjects grouped according to their obesity and psychiatric status into non-obese subjects with anxiety/mood disorders, non-obese subjects with no mental disorders, obese subjects with anxiety/mood disorders and obese subject with no mental disorders (*n* = 30 per group). The grouping of participants was subsequently checked using a structured psychiatric assessment 2 weeks prior to surgery.

All participants were recruited voluntarily based on participation criteria for the study:

*Inclusion criteria* included the following: Subjects 18 or over on the bariatric or abdominal laparoscopic surgery waiting list, willing to offer adipose tissue samples and giving written informed consent.

*Exclusion criteria* for the recruitment of participants were diagnosis of type 2 diabetes treated with insulin, acute or chronic inflammatory diseases, infectious diseases or cardiovascular diseases in the 6 months prior to participation in the study on account of the potential effect of these diseases or pharmacological treatments on inflammatory signals. Subjects diagnosed with both anxiety and mood disorders together or diagnosed with other mental disorders distinct to anxiety and mood disorders were also excluded.

Finally, the definitive sample consisted of non-obese subjects with anxiety/mood disorders (*n* = 31), non-obese subjects with no mental disorders (*n* = 25), obese subjects with anxiety/mood disorders (*n* = 34) and obese subjects with no mental disorders (*n* = 19). Psychiatric characteristics according to obesity are shown in Table S3.

### Ethics statement

Written informed consent was obtained from each participant. The present study and protocols were approved by the Ethics Committee of the Hospital Regional Universitario de Málaga and Hospital Universitario Virgen de la Victoria (PI10/01947) in accordance with the ‘Ethical Principles for Medical Research Involving Human Subjects’ adopted in the Declaration of Helsinki by the World Medical Association (64th WMA General Assembly, Fortaleza, Brazil, October 2013), Recommendation No. R (97) 5 of the Committee of Ministers to Member States on the Protection of Medical Data (1997), and the Spanish Data Protection Act (Ley Orgánica 15/1999 de Protección de Datos, LOPD).

### Sample collection

Venous blood was extracted into 10 mL K2-EDTA tubes (BD, Franklin Lakes, NJ, USA) and immediately processed to obtain serum, prior to the start of the surgical procedure and anesthesia induction. During the surgery, approximately 500 mg biopsies of subcutaneous (SAT) and visceral adipose (VAT) tissue were obtained from the same anatomical area at the beginning of the surgical procedure: SAT samples from the abdominal wall and VAT samples from the greater omentum. All of the samples were stored immediately at 80 °C.

### Psychiatric evaluation

Psychiatric evaluations were conducted 2 weeks prior to surgery and consisted of a structured psychiatric interview using the Spanish version of ‘International Mini Neuropsychiatric Interview’, version 5.0.0 (MINI), which was administered by clinical psychologists, specialized in clinical diagnostic interviews^46^. The MINI is a brief high structured interview that is compatible with the diagnostic criteria established by the ‘Diagnostic and Statistical Manual of Mental Disorders IV edition, text revision’ (DSM-IV-TR) and the ‘International Classification of Diseases’ (ICD-10). The MINI is structured in modules that explore common disorders of Axis I of the DSM-IV, as well as the risk of suicide and anti-social personality disorder^46^. The MINI interview has an approximate duration of 20 min and has been proven to have satisfactory reliability. In the present study, we selected patients with no mental disorders according to the MINI [i.e., negative for 16 (A to P) modules assessing the major Axis I psychiatric disorders in DSM-IV and ICD-10], patients with anxiety disorders (modules: E. panic disorder, F. agoraphobia, G. social phobia, H. obsessive-compulsive disorder, I. posttraumatic stress disorder and O. generalized anxiety disorder) and mood disorders or episodes (modules: A. major depressive episode, B. dysthymia, C. suicidality, D. manic or hypomanic episode)^47,48^.

The prevalence of psychotropic medication use during the last year was also determined.

### Biochemical determinations in serum samples

The participants’ serum samples were assessed for biochemical parameters in duplicate [glucose, total cholesterol, high-density lipoprotein (HDL), low-density lipoprotein (LDL), and triglycerides] and were measured by standard enzymatic methods. Insulin was analyzed by an immunoradiometric assay (BioSource International, Camarillo, CA, USA). HOMA-IR was calculated from fasting insulin and glucose with the following equation: HOMA-IR = fasting insulin (µIU/mL) x fasting glucose (mol/L)/22.5^49^. IL-1β, IL-6 and TNFα concentrations (pg/mL) were measured in serum with commercial enzyme-linked immunosorbent assay (ELISA) kits following the manufacturer’s instructions (Abcam plc, Cambridge, UK).

### Extraction of mRNA from adipose tissue biopsies and real-time PCR

Gene expression was assessed by real time-quantitative (RT-q) PCR after mRNA extraction. Total RNA was isolated from adipose tissue samples using the Trizol RNA isolation method (Invitrogen, Carlsbad, CA, USA) and purified with the RNeasy Lipid kit (QIAGEN, Valencia, CA, USA). RNA was reverse transcribed using Transcriptor Reverse Transcriptase kit (Roche Diagnostic, Barcelona, Spain). The amplifications were performed using a MicroAmp^®^ Optical 96-well reaction plate (Applied Biosystems, Barcelona, Spain) on an ABI 7500 Fast Real-Time PCR System (Applied Biosystems, Barcelona, Spain). RT qPCR reactions were carried out for the following genes using specific TaqMan^®^ Gene Expression Assays: *ADIPOQ*, *LEP*, *IL6, IL1B, TNF, CCL2, CSF3, ITGAM*, and *PLAUR*. During PCR, the Ct values for each amplified product were determined using a threshold value of 0.1. The specific signals were normalized by constitutively expressed cyclophilin (*PPIA*) signals using the formula 2^−ΔCt^.

### Statistical analysis

All data in the tables are expressed as number and percentage of subjects [*n* (%)], mean and standard deviation (mean** ± **SD) or mean and 95% confidence interval [mean (95% CI)]. The significance of differences in categorical variables was determined by using Fisher’s exact test or Chi-square test; while continuous variables were evaluated using Student’s t-test or the Mann-Whitney U test.

Relative mRNA levels of genes in the VAT and SAT were analyzed using multivariate and univariate analysis of covariance (ANCOVA) as a general linear model to indicate the relative effect of independent variables (factors) [i.e., obesity status (non-obese and obese groups), sex (women and men), mental disorder status [non-mental disorders (non-MD) and anxiety/mood disorders (A/MD) subgroups], type of mental disorder [non-MD, anxiety disorders (ANX) and mood disorders (MOOD) subgroups] and their interactions on mRNA expression, and controlling for covariates (i.e., age, BMI and psychiatric medication)^50^. Because this is an observational research design, we used the term ‘association’ to describe effects of independent variables (or interaction) with dependent variables in the ANCOVA models. *Post hoc* tests (Sidak correction) were used to compare subgroups. To ensure statistical assumptions for positively skewed distributions, we used log(10)-transformation of mRNA levels for all genes. Estimated marginal means and (95% CI) of mRNA levels were presented in the figures after back-transformation.

Correlation analyses were performed using the Pearson’s correlation coefficient (r) and Benjamini and Hochberg procedure was employed for correction of multiple comparisons.

Thresholds of 0.05 were applied for *P*-values. Statistical analyses were performed using Graph-Pad Prism version 5.04 (GraphPad Software, San Diego, CA, USA) and IBM SPSS Statistical version 22 (IBM, Armonk, NY, USA) softwares.

## Electronic supplementary material

Tables S1, S2 and S3
