# Supplementary material for: Inflammatory gene expression in adipose tissue according to diagnosis of anxiety and mood disorders in obese and non-obese subjects
Source: Sci Rep. 2018 Nov 30;8:17518. doi: 10.1038/s41598-018-35759-9 (PMC6269530; doi:10.1038/s41598-018-35759-9)
Supplement: Supplementary file 1 — Tables S1, S2 and S3 [file 41598_2018_35759_MOESM1_ESM.doc]

**Inflammatory gene expression in adipose tissue according to diagnosis of anxiety and mood disorders in obese and non-obese subjects**

***Running title: Adipose tissue inflammation, obesity and mental disorders***

Leticia Coín Aragüez^1*^, Francisco Javier Pavón^2*^, Alba Contreras^2*^, Adriana-Mariel Gentile^3^, Said Lhamyani^3^, Yolanda De Diego-Otero^2^, Yolanda Casado^2^, Wilfredo Oliva Olivera^1^, Gabriel Olveira^4^, Francisco J. Tinahones^1^, Lucía Pérez Costillas^2🖂^, Rajaa El Bekay^5🖂^

**^1^**Unidad de Gestión Clínica Endocrinología y Nutrición, Instituto de Investigación Biomédica de Málaga (IBIMA), Complejo Hospitalario de Málaga (Virgen de la Victoria), Universidad de Málaga. CIBER de Fisiopatología de la Obesidad y Nutrición (CIBERobn), Instituto de Salud Carlos III, Spain.

^2^Unidad de Gestión Clínica de Salud Mental, Instituto de Investigación Biomédica de Málaga (IBIMA), Hospital Regional Universitario de Málaga, Universidad de Málaga, Málaga , Spain.

^3^IBIMA. Universidad de Málaga. Facultad de Ciencias. Campus Teatinos s/n - 29071 - Málaga, Spain.

^4^UGC Endocrinología y Nutrición, Instituto de Investigación Biomédica de Málaga (IBIMA), Hospital Universitario Regional de Málaga, Universidad de Málaga. CIBERDEM CB07/08/0019, Instituto de Salud Carlos III, Spain.

^5^Unidad de Gestión Clínica Endocrinología y Nutrición, Instituto de Investigación Biomédica de Málaga (IBIMA), Hospital Regional Universitario de Málaga, Universidad de Málaga. CIBER de Fisiopatología de la Obesidad y Nutrición (CIBERobn), Instituto de Salud Carlos III (ISCIII), Spain.

**(*****)** These authors contributed equally to the present study.

**(**^🖂^**) Corresponding Authors:**

Dr. Rajaa El Bekay ([elbekay@gmail.com](mailto:elbekay@gmail.com))

Dr. Lucía Pérez Costillas ([lpcostillas@gmail.com](mailto:lpcostillas@gmail.com))

**Table S1.** Correlation analysis between relative mRNA expression of genes in the visceral (VAT) and subcutaneous (SAT) adipose tissues of non-obese and obese patients.

| ***Gene***  **(Protein)** | **VAT and SAT** | | | |
| --- | --- | --- | --- | --- |
|  | **Non-obese group**  **(BMI<30)**  **n=56** | | **Obese group**  **(BMI≥30)**  **n=53** | |
|  | **Correlation coefficient (r)^a^** | ***P*-value** | **Correlation coefficient (r) ^a^** | ***P*-value** |
| ***ADIPOQ* (Adiponectin)** | - | ns | - | ns |
| ***LEP* (Leptin)** | +0.495 | **0.001 ^b^** | - | ns |
| ***IL6* (Interleukin 6/IL6)** | +0.563 | **<0.001 ^b^** | +0.391 | **0.004 ^b^** |
| ***IL1B* (Interleukin 1 Beta/IL1β)** | +0.488 | **<0.001 ^b^** | +0.343 | **0.012 ^b^** |
| ***TNF* (Tumor Necrosis Factor Alpha/TNFα)** | +0.345 | **0.011 ^b^** | +0.308 | 0.025 |
| ***CCL2* (Chemokine (C-C motif) Ligand 2/CCL2)** | +0.292 | 0.032 | +0.283 | 0.040 |
| ***CSF3* (Colony Stimulating Factor 3/CSF3)** | - | ns | - | ns |
| ***ITGAM* (Integrin, Alpha M/ITGAM)** | +0.289 | 0.034 | +0.435 | **0.001 ^b^** |
| ***PLAUR* (Plasminogen Activator, Urokinase Receptor/PLAUR)** | - | ns | +0.370 | **0.006 ^b^** |
| (^a^) All variables were normally distributed. Spearman correlation coefficient (rho) was used for correlation analysis.  (^b^) Adjusted *P*-values were calculated for Holm-Bonferroni correction.  Abbreviations: ns, non-significant. | | | | |

**Table S2.** Psychiatric characteristics in non-obese and obese patients.

| **Variables** | | **Non-obese group**  **(BMI<30)** | **Obese group**  **(BMI≥30)** | ***P*-value** |
| --- | --- | --- | --- | --- |
|  |  | **n=56** | **n=53** |  |
| **Mental disorders**  **[n (%)]** | No | 25 (44.6) | 19 (35.8) | 0.475 |
|  | Anxiety disorders | 22 (39.3) | 21 (39.6) |  |
|  | Mood disorders | 9 (16.1) | 13 (24.5) |  |
| **Psychiatric medication**  **[n (%)]** | No | 18 (32.1) | 16 (30.2) | 0.839 |
|  | Anxiolytics | 34 (60.7) | 30 (56.6) |  |
|  | Antidepressants | 24 (42.9) | 28 (52.8) |  |
|  | Neuroleptics | 5 (8.9) | 5 (9.4) |  |
| (^a^) *P*-value from Fisher's exact test or Chi-square test | | | | |

**Table S3. Psychiatric characteristics according to obesity.**

| **Variables** | | | **Non-obese group**  **(BMI<30)** | **Obese group**  **(BMI≥30)** | ***P*-value** |
| --- | --- | --- | --- | --- | --- |
|  |  |  | **n=56** | **n=53** |  |
| **Psychiatric status**  **[n (%)]** | No mental disorders | | 25 (44.6) | 19 (35.8) | 0.475 ^a^ |
|  | Mental disorders | Anxiety disorders | 22 (39.3) | 21 (39.6) |  |
|  |  | Mood disorders | 9 (16.1) | 13 (24.5) |  |
| **Psychiatric medication**  **[n (%)]** | No | | 18 (32.1) | 16 (30.2) | 0.839 ^a^ |
|  | Anxiolytics | | 34 (60.7) | 30 (56.6) |  |
|  | Antidepressants | | 24 (42.9) | 28 (52.8) |  |
|  | Neuroleptics | | 5 (8.9) | 5 (9.4) |  |
| (^a^) *P*-value from Fisher's exact test or Chi-square test  Abbreviations: BMI, Body Mass Index | | | | | |
